# Primary female genital system lymphoma complicated by a recurrent mucinous borderline ovarian tumor: a case report and review of the literature

**DOI:** 10.1186/s12905-021-01568-y

**Published:** 2021-12-18

**Authors:** Erqiu Du, Xiangdong Qu, Wei Xu, Hongsheng Lu

**Affiliations:** 1 Department of Obstetrics and Gynecology, Taizhou Central Hospital, (Taizhou University Hospital), No. 999, Donghai Avenue, Taizhou Economic Development Zone, Taizhou, Zhejiang Province China; 2 Department of Pathology, Taizhou Central Hospital, (Taizhou University Hospital), No. 999, Donghai Avenue, Taizhou Economic Development Zone, Taizhou, Zhejiang Province China

**Keywords:** Non-Hodgkin's lymphoma, Primary female genital system lymphoma, Uterine lymphoma, Extranodal lymphoma, Mucinous borderline ovarian tumor

## Abstract

**Background:**

Primary female genital system lymphoma (PFGSL) is an infrequent entity. All genital organs may be affected, and most PFGSLs are localized to the cervix, uterine body, and ovaries. The clinical manifestations are nonspecific, which complicates a timely diagnosis. We report an unexpected case of PFGSL and discuss the disease characteristics by reviewing the literature.

**Case presentation:**

A 48-year-old G3/P2 woman presented to the Department of Gynecology with a physical examination. Ultrasound examination and CT revealed pelvic masses. The woman underwent surgical treatment because of the pelvic masses and underwent a hysterectomy for a recurrent mucinous borderline ovarian tumor. However, the results of the postoperative pathological examination showed diffuse large B-cell lymphoma of the endometrium. After four courses of chemotherapy, the woman was in good condition. The clinical manifestations were nonspecific, which made a timely diagnosis complex.

**Conclusion:**

This case highlights the importance of the difficulty in detecting early PFGSL early and how easily nonspecific manifestations can be ignored. It may lead to missing the best time for early treatment.

## Background

Primary female genital system lymphoma (PFGSL) is an infrequent entity and constitutes 0.2–1.1% of all extranodal lymphoma cases. In the U.S., there are an estimated 165 new cases of PFGSL annually [1], but in China, PFGSL is an extremely rare cause of female genital tumors, and few cases have been reported. All genital organs may be affected, and most PFGSLs are localized to the cervix, uterine body, and ovaries. Lymphoma of the endometrium is exceedingly rare. If the clinical manifestation is nonspecific, it can be challenging to make a timely diagnosis. In the last year, we treated a case of PFGSL at Taizhou General Hospital of Taizhou University. It was found by postoperative pathological examination for the hysterectomy of a recurrent mucinous borderline ovarian tumor. This case revealed that early PFGSL detection is challenging and its symptoms are easy to ignore. Herein, the authors report a case of a woman with asymptomatic PFGSL with a recurrent borderline ovarian tumor.

## Case presentation

**Fig. 1:**
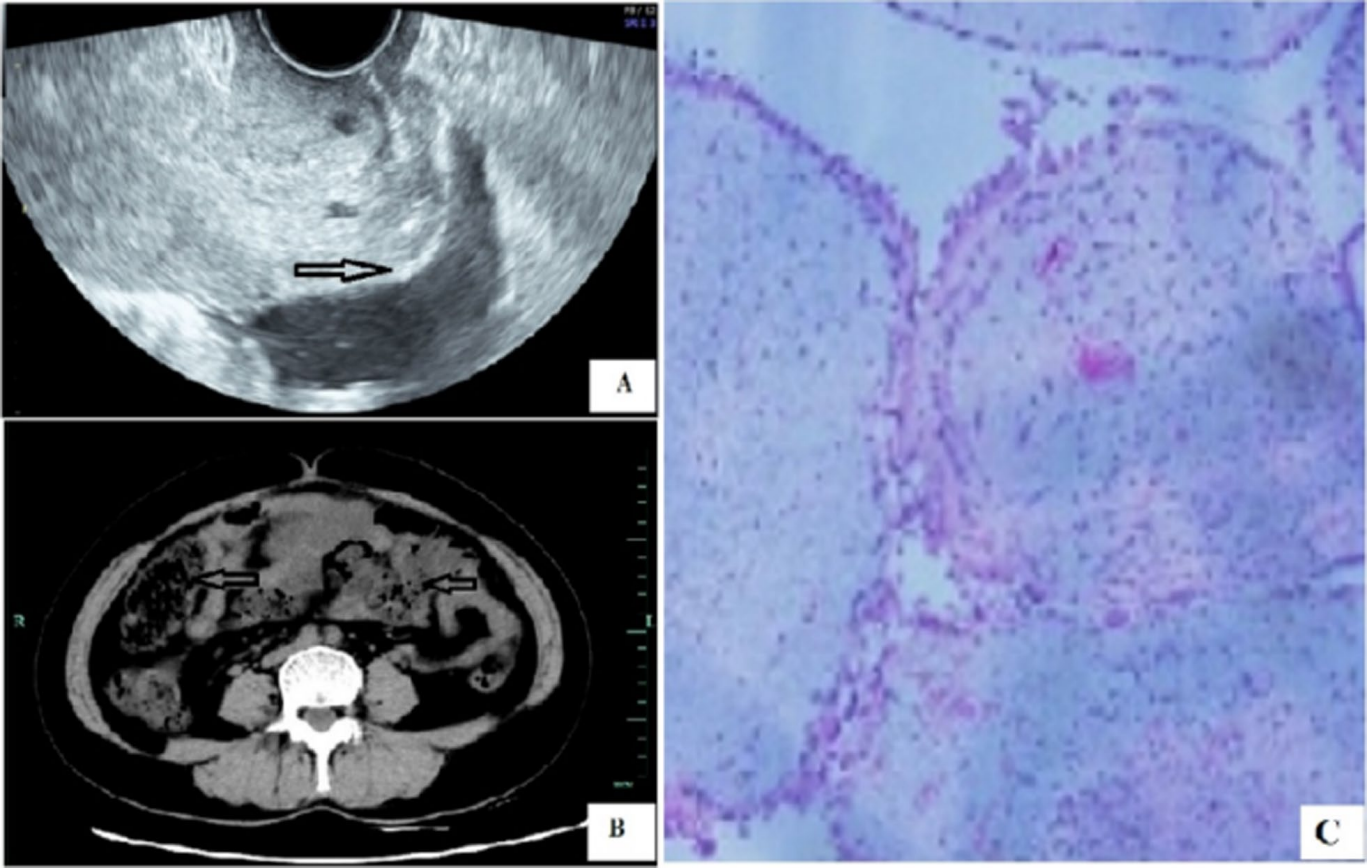
The size of the pelvic mass was 15.8 × 12.5 × 11.7 cm^3^ and 6.4 × 5.8 × 6.9 cm^3^ by ultrasonography (**A**). The size of the pelvic mass was 14.5 × 10 cm^2^ and 6.5 × 6.4 cm^2^ by enhanced CT (**B**). The pelvic mass was confirmed to be a borderline ovarian tumor by HE staining analysis (**C**)

**Fig. 2:**
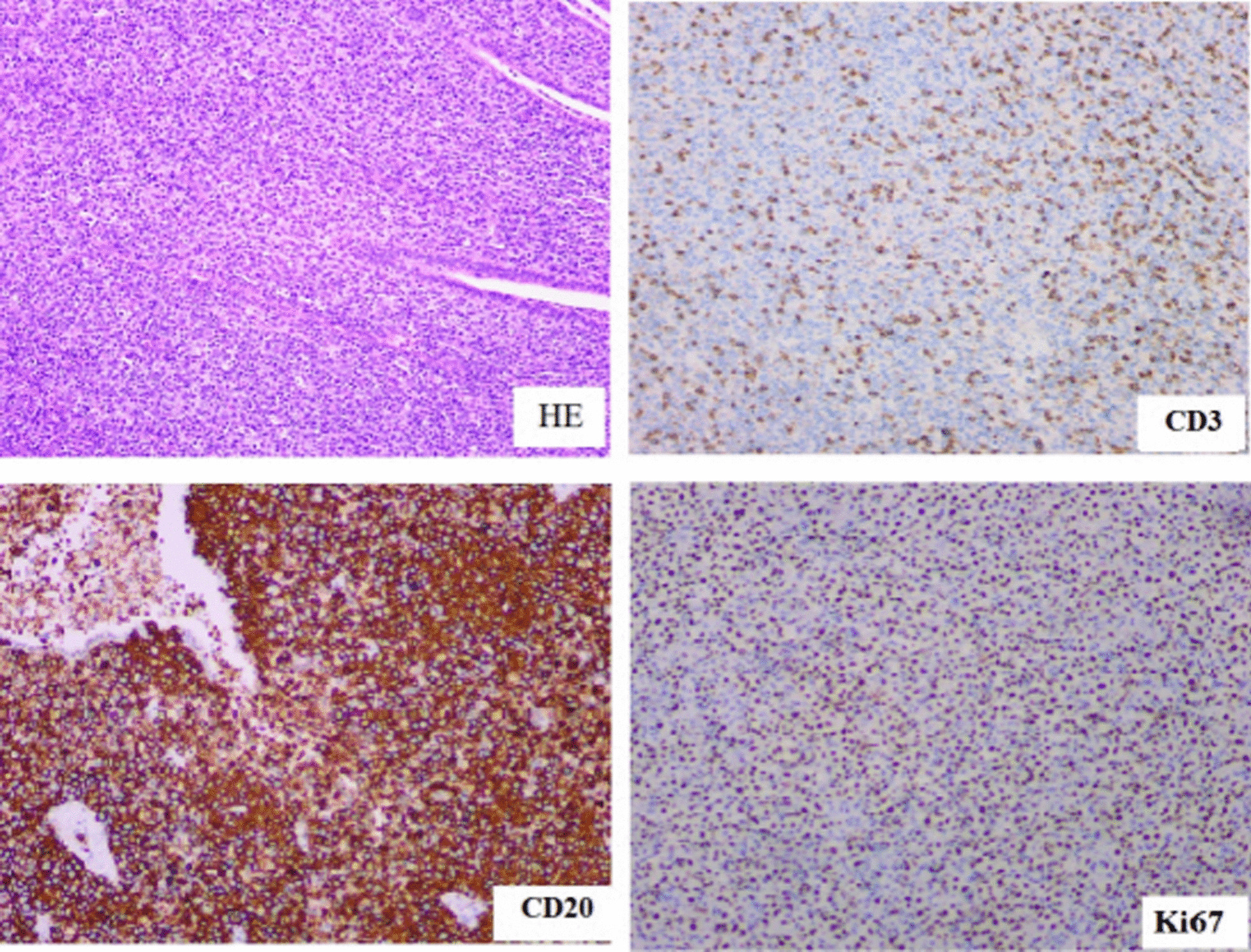
Hematoxylin–eosin (HE) staining and immunohistochemistry were carried to analyze the biopsy specimen of the case. HE staining revealed substantial growth of tumor cells, which had small and round nuclei containing conspicuous nucleoli. Mitotic figures were identified. Immunohistochemical staining revealed positive staining for CD3, Ki67, and CD20

A 48-year-old G3/P2 woman presented to the Department of Gynecology with a physical examination. Ultrasonography (Fig. 1A) and enhanced computed tomography (ECT) (Fig. 1B) revealed pelvic masses. She was recommend to undergo laparoscopic ovarian cystectomy for a borderline ovarian tumor eight years before. During the eight years, she did not have regular medical examinations because there were no symptoms of diseases. She chose to undergo surgical treatment for pelvic masses. Finally, she underwent a hysterectomy and pelvic lymph node dissection for a recurrent mucinous borderline ovarian tumor (Fig. 1C). Postoperative routine examination showed endometrial lymphoma, and the other lymph nodes were not involved. Testing for immunoglobulin heavy chain (IGH) gene rearrangement showed a positive result (Table 1). Uterine lymphoma was derived from the primary female genital system and was diagnosed as non-Hodgkin's lymphoma, consistent with diffuse large B-cell non-Hodgkin's lymphoma. Hematoxylin–eosin (HE) staining and immunohistochemistry were carried out to analyze the case specimen (Fig. 2). The patients underwent bone marrow biopsy and PET-CT to observe whether there were other lymph node abnormalities. However, there was no evidence of bone marrow involvement by microscopic examination, and there was no abnormality in the whole body scan by PET-CT. Currently, the patient has received four postoperative courses of CHOP chemotherapy in the Department of Hematology. To date, no abnormality has been found in the follow-up.

**Table 1:** The result of gene rearrangement

Gene rearrangement	Gene frame region	Result
IGH	Tube A(FR1-JH)	Positive
	Tube B(FR2-JH)	Positive
	Tube C(FR3-JH)	Positive
	Tube D(DH-JH)	Positive
	Tube E(DH7-JH)	Negative
IGK	Tube A(Vk-Jk)	Positive
	Tube B(Vk-Kde + intron-Kde)	Negative
IGL	Vλ-Jλ	Negative

## Discussion and conclusions

PFGSL is a sporadic neoplastic disorder that accounts for 1.5% of all non-Hodgkin's lymphomas. PFGSL usually occurs in women older than 60 years of age [1, 2]. The most commonly affected genital locations are the ovaries and uterus; the vagina and vulva are rarely affected. However, lymphoma of the endometrium is exceedingly rare. Its clinical manifestations consist of abnormal vaginal bleeding and discharge, abdominal pain, and abdominal masses. The primary histological type of PFGSL is B-cell lymphoma, of which diffusing large B-cell lymphoma (DLBCL) is the most frequent subtype [3]. The diagnosis of PFGSL must meet the following criteria: (i) the disease process is clinically confined to the uterus at the time of the initial diagnosis; (ii) a full investigation fails to reveal any evidence of disease elsewhere in the body; (iii) there is a lack of abnormal cells in the bone marrow or peripheral blood; and (iv) if further lymphomatous deposits occur at sites that are removed from the genital tract, a time interval of at least several months should elapse between the appearance of the primary and secondary tumors [4, 5]. Therefore, combined with the characteristics of this case, the patient described in this report was diagnosed with DLBCL of PFGSL arising in the endometrium.

Previous case series have reported that DLBCL is the most common histological type of PFGTL [3, 6]. Its clinical symptoms are usually nonspecific and include vaginal bleeding (70%), perineal discomfort (40%), and persistent vaginal discharge (20%) [7]. In the case described in this report, genital symptoms and abnormal manifestations on an imaging examination were not observed. Therefore, the condition was difficult to diagnose by cytology, cervical biopsy, and endometrial curettage. We obtained malignant lymphoma tissue in the deep endometrial stroma and diagnosed the patient using a transcervical needle biopsy [8].

There are currently no randomized clinical trials or specific guidelines addressing the treatment of PFLGT. The patients receive comprehensive treatment, which usually involves a combination of surgery, chemotherapy, and radiotherapy. Surgical treatment aims to define the pathology and stage of the tumor, reduce the tumor load, and improve the efficacy of postoperative chemotherapy or radiotherapy. At present, comprehensive treatment based on chemotherapy and surgery can improve the survival rate of patients. However, chemotherapy is mainly administered for lymphoma. Regarding rituximab, cyclophosphamide, doxorubicin, vincristine,and prednisone(R-CHOP) therapy, the combination of CHOP therapy, the standard treatment, and rituximab, a chimeric anti-CD20 monoclonal immunoglobulin G antibody, has been successfully used to treat CD20-positive B-cell non-Hodgkin's lymphoma [9]. At present, R-CHOP therapy is the first-line treatment for diffuse large B-cell lymphoma. R-CHOP therapy is composed of rituximab, cyclophosphamide, doxorubicin, vincristine, and prednisone. It has been reported that the efficacy rate of R-CHOP therapy for DLBCL in the female reproductive system can reach 71.88% after surgery and chemotherapy [10]. However, there is no standard treatment for peripheral T-cell lymphoma. The options include CHOP therapy and gemcitabine combined with L-asparaginase. The chemotherapy course should be 4–6 cycles and 2–3 periods should occur after complete remission [11, 12]. Because of the disease's unusual nature, the clinical manifestations are nonspecific, the diagnosis is challenging, and the treatment method is entirely different from those for other reproductive system tumors. Early lymphoma analysis and the pathological type and stage of lymphoma can allow PFGSL patients to receive timely and accurate treatment and improve patient survival rates. The clinical symptoms of PFGSL are not typical, and the prognosis is poor. Therefore, clinicians should strengthen the analysis and understanding of the disease and attach great importance to the pathological and immunohistochemical results, early diagnosis of lymphoma, and pathological type and staging of lymphoma so that patients with PFGSL can receive timely and accurate treatment and that the survival rate of patients can be improved.

## Data Availability

The datasets used during the current study are available from the corresponding author on reasonable request. Department of pathology, Taizhou General Hospital, (Taizhou University Hospital), Taizhou, Zhejiang, China.

## Abbreviations

DLBCL: Diffusing large B-cell lymphoma
PFGSL: Primary female genital system lymphoma
CT: Computed X-ray tomography;
ECT: Enhanced computed X-ray tomography
IGH gene: Immunoglobulin heavy chain gene
HE staining: Hematoxylin–eosin staining
R-CHOP: Rituximab, cyclophosphamide, doxorubicin, vincristine, and prednisone
